# The lived experiences of female only-child caregivers in transnational settings

**DOI:** 10.1186/s12905-026-04548-2

**Published:** 2026-05-25

**Authors:** Zihan Dang, Charles E Basch

**Affiliations:** Department of Health Studies and Applied Educational Psychology, Columbia University, 525 West 120th Street, New York, NY 10027 USA

**Keywords:** Transnational caregiving, Only-child caregiver, Female caregiver

## Abstract

**Background:**

China’s One-Child Policy created only-child daughters solely responsible for aging parents. For those abroad, caregiving is further complicated by distance, immigration rules, and cultural expectations. Little is known about how these women manage cross-border caregiving.

**Objectives:**

This study examines the experiences of only-child Chinese daughters in the United States as they care for aging parents across borders.

**Methods:**

Guided by the Meaning-Making Model, we conducted semi-structured interviews with 60 married, only-child daughters aged 30–45. Each had provided remote care for at least one year. Data were analyzed using reflexive thematic analysis.

**Results:**

Three central findings emerged. First, caregivers redefined what it means to be present, capable, and filial, compensating for physical distance with increased attentiveness, emotional availability, and reciprocal support. Second, these women experienced and managed conflicting emotions, balancing their personal goals with deeply held filial expectations. Third, uncertainty and anticipatory grief strongly influenced their decisions on traveling, career advancement, citizenship choices, and care arrangements, highlighting how planning for future needs is an ongoing, emotionally charged process.

**Conclusions:**

Transnational caregiving among only-child Chinese daughters is both emotionally complex and shaped by cultural expectations. This study introduces *anticipatory caregiving* to describe the future-oriented emotional and logistical work that defines long-distance care. These insights show that transnational caregiving is not just a set of tasks. It is a unique psychological and cultural experience that demands new, tailored support.

**Supplementary Information:**

The online version contains supplementary material available at 10.1186/s12905-026-04548-2.

## Introduction

Informal caregiving is among the most emotionally demanding and health-relevant forms of unpaid labor worldwide. Unlike professional care, it arises within intimate family relationships and is driven by affection, moral duty, and cultural obligation rather than financial reward [22]. Globally, most care needs are met by unpaid caregivers—equivalent to approximately two billion individuals working full-time without remuneration, representing nearly 5% of the world’s gross domestic product [21]. Despite its considerable social and economic contribution, informal caregiving remains largely unrecognized in policy and research frameworks [31].

Caregiving is also profoundly gendered. Women provide an estimated 80% of unpaid care worldwide, often while simultaneously maintaining paid employment and family responsibilities [8]. IIn China, compared with males, female caregivers perform 71.6% of unpaid care work, dedicating approximately three times more hours to caregiving [20]. Managing both professional and domestic responsibilities generates potential for a constant overlap between productive and reproductive labor, placing women at heightened risk of psychological strain and physical health decline [3].

Recent demographic analyses suggest that approximately 60 million women in contemporary China shoulder a caregiving responsibility score exceeding 0.8, an indicator equivalent to providing care for at least one elderly family member experiencing significant health limitations and lacking sibling assistance [24]. This imbalance underscores that informal female caregiving is shaped not only by family affection and moral duty but also by structural inequities that disproportionately assign women emotional and domestic labor, leading to cumulative psychological stress and adverse health consequences.

These inequities take on a deeper cultural and demographic dimension in China, where the convergence of rapid population aging, the long-term effects of population control policies, and enduring family traditions has produced a distinctive and historically specific caregiving landscape. The One-Child Policy (1979–2015) fundamentally restructured family organization by eliminating sibling networks and concentrating filial responsibilities onto a single offspring [13]. Women from this generation occupy a uniquely complex sociocultural position: while benefiting from expanded educational and professional opportunities, they remain influenced by enduring Confucian ideals of filial piety (*xiao*), which emphasize respect, devotion, and moral responsibility toward parents [2]. The coexistence of modern gender equality and traditional filial morality situates women at the intersection of competing expectations, striving for autonomy and achievement while sustaining deep intergenerational obligations.

For many only-child women now living abroad, these tensions are further magnified by transnational caregiving, which refers to providing care to aging parents while residing in a different country [18]. Unlike caregiving within the same household or local community, transnational caregiving requires navigating physical distance, time-zone differences, and limited direct contact, often relying on digital communication and remote coordination of financial, medical, and emotional support [1]. In the absence of siblings, these single-child daughters frequently bear exclusive responsibility for both the emotional and logistical aspects of parental care, while simultaneously managing spousal, parental, and professional roles in host societies. This convergence of roles produces an ongoing and multidimensional strain that blurs the boundaries among obligation, affection, and self-preservation.

Existing research on caregiving highlights this dual emotional terrain. On one hand, caregiving is consistently linked to negative psychological outcomes, including chronic stress [33] and depressive symptoms [27]. On the other hand, many caregivers report feelings of happiness associated with successfully managing caregiving challenges [10], perceptions of personal growth [4], and fulfillment through giving care [23]. This coexistence of strain and reward suggests that caregiving should not be viewed as a static state of burden or benefit, but rather as a dynamic psychological process involving continual evaluation, emotional fluctuation, and the redefinition of meaning.

Recent scholarship has moved away from outcome-focused explanations and toward process-based frameworks that highlight how caregivers interpret and adjust to ongoing stressors. Within this shift, meaning-making becomes a key mechanism through which caregivers navigate conflicting emotions and maintain psychological balance over time. The Meaning-Making Model [30], rooted in stress and coping theory, views adaptation as an interpretive process in which individuals work to maintain coherence between situational meaning, their appraisal of a specific stressful event, and global meaning, or the broader beliefs, values, and life goals that shape their understanding of the world. When these two levels of meaning diverge, individuals engage in cognitive and emotional reappraisal to restore alignment and preserve a stable sense of purpose and identity.

Despite expanding scholarship on caregiving, three important gaps remain. First, much of the existing work treats caregiving outcomes as either negative or positive, which obscures the fluid, overlapping, and continually reconstructed nature of caregivers’ emotional experiences. Second, little attention has been given to the psychological meaning-making processes that shape how caregivers interpret, reframe, and negotiate their experiences within cultural and transnational contexts, especially in situations marked by prolonged uncertainty. Third, transnational caregiving in only-child families, where filial responsibility is concentrated, and social support is limited, has not been adequately examined in terms of how women rebuild meaning, sustain coherence, and maintain psychological balance across cultural and structural divides.

This study addresses these knowledge gaps by examining the lived experiences of female only-child caregivers from China’s One-Child Generation who are currently residing in the United States. The purpose of this study is to generate an in-depth and contextually grounded understanding of the meanings, emotional complexities, and caregiving negotiations embedded within transnational caregiving experiences. By centering participants’ narratives and lived realities, this study contributes to the qualitative literature on migration, caregiving, and family relationships by illuminating how cultural expectations, gendered responsibilities, and emotional reciprocity are interpreted and navigated across geographical distance.

## Method

This study comprised single, semi structured interviews with adults who self-identified (a) an ethnically Chinese woman socialized with traditional filial values; (b) an only child with no biological, adopted, or half-siblings; (c) aged 30–45; (d) residing in the United States, with at least one living parent in China; (e) engaged in remote caregiving for more than 12 months and continuing at the time of data collection; and (f) English proficiency adequate for a 60-minute interview.

The researcher employed both purposive and snowball sampling. Purposive sampling enabled the intentional selection of participants who met the study’s inclusion and exclusion criteria, ensuring the sample was relevant to the research questions. Snowball sampling facilitated the identification of additional participants through referrals from initial recruits. Recruitment was conducted through online forums and communities widely used by Chinese-speaking populations in the United States, including 1Point3Acres, a prominent platform for Chinese-speaking professionals, and Rednotes, a community for cultural and social engagement among Chinese expatriates.

All procedures performed in this study involving human participants were conducted in accordance with the ethical standards of the Institutional Review Board (IRB), and with the 1964 *Declaration of Helsinki* and its later amendments. Informed consent was obtained from all participants prior to participation. Interviews were conducted via Zoom from October 2024 to September 2025 in English, lasting approximately 60 min each. Interviews were audio-recorded, transcribed, and deidentified before analysis.

The interview guide (see Supplementary Material 1), developed using the Meaning-Making Model, explored the emotional, cultural, and structural dimensions of caregiving across distance. Questions addressed perceived burdens and rewards, caregiving beliefs and moral traditions, emotional responses, and evolving perceptions of caregiving identity. Prompts specifically examined (1) situational meaning (caregiving stressors), (2) global meaning (values, filial beliefs, and moral frameworks), and (3) reappraisal and coherence (processes of reconstructing meaning over time). To enhance interpretive accuracy, participants reviewed summarized bullet points through member check-in for confirmation or clarification.

All 60 interviews were coded by one analyst. Data Analysis was conducted using NVivo 14 following Braun and Clarke’s [7] six phases of reflexive thematic analysis: familiarization, coding, theme generation, theme review, defining themes, and reporting. Coding followed an abductive logic integrating inductive insights from participants’ narratives with deductive sensitization from the Meaning-Making framework. Line-by-line codes were iteratively refined, clustered into categories, and inductively developed into subthemes, which were then synthesized into major themes and vetted through constant comparison to ensure coherence, distinctiveness, and theoretical alignment [29]. Verbatim quotations were used strategically to substantiate analytic claims while ensuring the narrative remained interpretive rather than descriptive [11]. To enhance analytic rigor, the researcher maintained an audit trail documenting coding decisions, theme development, and analytic reflections. Interpretations were further strengthened through iterative engagement with the data, comparison across cases, and ongoing discussions with co-authors to challenge assumptions and refine thematic boundaries beyond member checking.

### Author positionality

The author is a Chinese-only child engaged in long-distance caregiving for aging parents, sharing cultural values and lived experiences with the study participants. This positionality provided unique insight into participants’ perspectives, particularly regarding filial obligations, emotional experiences, and caregiving practices. To ensure rigor and minimize bias, the researcher engaged in reflexive practices, including journaling, memo writing, and continuous self-examination of assumptions throughout the analytic process. Post-interview member check-ins on summarized findings were conducted to confirm resonance with participants’ experiences. In addition, reflexivity and positional awareness were integrated into the analytic process to critically evaluate how the researcher’s background may shape interpretation, thereby enhancing transparency, credibility, and depth of analysis.

### Ethical considerations

This qualitative study received Institutional Review Board approval from Teachers College, Columbia University (25–046).

## Results

As indicated in Table 1, the study comprised 60 participants aged 30 to 45 years, with the largest proportion (43%) aged 30 to 32 years. Most participants’ mothers were aged 55 to 65 years, while fathers were generally older, with the majority aged 59 years or older. Nearly half of the participants (47%) had provided care for 4 to 6 years, followed by 23% for 1 to 3 years and 30% for 7 to 10 years. The majority reported annual household incomes between $50,000 and $150,000.

**Table 1:** Participant and Parent Characteristics (*N* = 60)

Category	Range	*n* / %
Participant Age (yrs)	30–32	26 (43%)
	33–34	17 (28%)
	35–37	14 (23%)
	38–40	3 (6%)
Years of Caregiving	1–3	14 (23%)
	4–6	28 (47%)
	7–10	18 (30%)
Number of Dependents	1	9 (15%)
	2	46 (77%)
	3–4	5 (8%)
Mothers’ Age (yrs)	55–59	26 (43%)
	60–65	26 (43%)
	66–70	8 (14%)
Fathers’ Age (yrs)	55–59	14 (23%)
	60–65	37 (62%)
	66–70	9 (15%)
Annual Household Income (USD)	0–50 K	8 (13%)
	50–150 K	39 (65%)
	≥ 200 K	13 (22%)
Employment Status	Full-time	47 (79%)

As indicated in Table 2, three major themes (T1–T3) emerged, capturing how participants defined and made sense of their lived experiences in transnational caregiving settings.

**Table 2:** Summary of Themes and Subthemes

Theme	Subthemes
Theme 1: Reconstructing Care Across Distance	1.1 Redefining Presence and Filial Practice Across Distance 1.2 Developing Capability and Resilience Through Distance 1.3 From Obligation to Reciprocity 1.4 Distance as Both Constraint and Resource
Theme 2: Moral Tension and Emotional Duality	2.1 Persistent Guilt and Moral Inadequacy 2.2 Migration as Both Achievement and Moral Conflict 2.3 Communication and Relational Strain
Theme 3: Navigating Uncertainty and Change	3.1 Living with Persistent Uncertainty and Anticipation 3.2 Anticipatory Grief and Emotional Pre-Loss 3.3 Life Adjustment

### Theme 1: reconstructing care across distance

#### Subtheme 1.1 redefining presence and filial practice across distance

Most participants described gradually redefining what it means to “be present” while caring for their parents across national borders. Physical distance, initially experienced as a rupture in filial duty, was often accompanied by feelings of anxiety, guilt, and helplessness. As one participant reflected, “When something happens, I’m always the last one to know. That distance makes me feel like I’ve already failed as a daughter before I even try.” However, over time, participants began to reinterpret distance not solely as a limitation, but as a condition that required new forms of connection, attentiveness, and responsibility.

As participants learned to coordinate medical appointments online, arrange home-based services through digital platforms, and maintain consistent emotional communication, their initial distress shifted toward a growing sense of competence, control, and psychological resilience. One participant explained, “I’m not physically in the room. But I’m basically behind every decision, you know…every appointment, every payment, every plan.” Another shared, “My day starts with checking their messages and ends with making sure everything is settled for them. It’s like I’m there, but, you know… I am just not physically manifested there.”

This transition reflects an important interpretive shift: caregiving was no longer understood primarily as physical co-presence, but as a multidimensional practice grounded in emotional availability, proactive coordination, and sustained moral commitment. As one participant articulated:

Filial piety, for me, is to provide sufficient care at my best…I know I can’t be physically there for my parents, but when I stay up all night arranging their travel plans and paying for their bills, I feel I’m fulfilling my duty, you know, as those staying with their parents.

These narratives suggest that participants were not merely adapting caregiving practices to geographical separation but actively reconstructing the meaning of filial responsibility itself. Rather than equating filial piety solely with physical proximity, participants reinterpreted care as an ongoing process of emotional vigilance, logistical coordination, and moral accountability. This shift reflects how transnational caregiving reshaped traditional understandings of presence, transforming caregiving into a psychologically mediated and technologically supported form of relational labor.

#### Subtheme 1.2 developing capability and resilience through distance

Participants’ narratives revealed a progression from initial helplessness to increasing competence and psychological resilience. Early caregiving experiences were often marked by uncertainty and a perceived loss of control. As one participant described, “At the beginning, it is like… everything felt out of my hands, I didn’t even know who to call or what to do when something happened.” However, through repeated engagement with logistical and emotional challenges, participants gradually developed new skills, confidence, and a sense of agency.

Over time, caregiving from a distance became a site of capability-building. Participants described learning to navigate healthcare systems remotely, coordinate complex arrangements, and manage their parents’ emotional needs, often simultaneously. One caregiver reflected, “Those two years turned me into a superwoman, I learned to solve almost everything, from handling hospital things to house renovation, and you know, I feel I even become a self-trained psychologist to support my parents emotionally.”

Importantly, participants no longer framed their actions solely as coping strategies but as evidence of personal growth and resilience. As one participant articulated, “It’s not just about solving problems anymore, you know, this entire experience changed how I see myself. I became someone my parents can rely on, even from far away. I kind of proud of myself.”

Participants’ accounts revealed that resilience was not described as an inherent personality trait, but rather as something gradually developed through repeated exposure to uncertainty, emotional strain, and caregiving demands. The caregiving process simultaneously generated stress and facilitated self-transformation, with many participants redefining themselves as more capable, emotionally adaptive, and resourceful over time. This contradiction highlights how long-distance caregiving functioned both as a source of vulnerability and as a context for identity reconstruction and personal growth.

#### Subtheme 1.3 from obligation to reciprocity

While caregiving from a distance often began as a moral obligation, many participants described how it gradually evolved into a reciprocal exchange of affection, gratitude, and emotional understanding. Rather than remaining a one-sided duty, caregiving was increasingly experienced as a relational process shaped by ongoing interaction and reflection. As one participant expressed, “At first, I felt like I had to do it because it was my responsibility. But over time, it became something we both give to each other, it’s not just me taking care of them anymore.”

Acts of giving, whether through financial support, meaningful gifts, or sustained emotional presence, were often described as symbolic gestures that expressed both gratitude and personal agency. These gestures bridged physical distance by making care visible and emotionally tangible. One participant reflected, “When I send something to them or help arrange everything from here, it’s my way of showing that I’m still part of their daily life.” Parental acknowledgment, in turn, reinforced caregivers’ sense of purpose and self-worth. As another participant noted,

My mom gave me everything when I was young…it is not really the duty to take care of her, it is, you know, the love…. I remember, I used my first month’s salary to buy my mom a golden bracelet… She was so happy, and she cried. I was motivated to earn more and buy her more things.

While caregiving initially emerged from culturally embedded expectations of filial obligation, many participants gradually reframed caregiving as an emotionally reciprocal relationship grounded in mutual appreciation, affection, and recognition. This reinterpretation appeared to soften the moral rigidity traditionally associated with filial duty, allowing participants to construct caregiving as an expression of relational connection rather than solely a culturally prescribed responsibility.

#### Subtheme 1.4 distance as both constraint and resource

Participants also recognized that distance could mitigate daily conflict and cultivate deeper emotional reciprocity. The reduction in day-to-day caregiving interactions allowed space for reflection, empathy, and more intentional communication. For example, one participant observed:

We used to fight every day, but now, you know, with distance, we don’t have time to fight. Our conversation is only about important topics. I feel I became more filial in her eyes after I moved to America…I make her less angry, and she’s more satisfied with my attitude.

Although physical distance generated guilt, uncertainty, and emotional strain, it also created emotional space that reduced everyday interpersonal conflict and facilitated more intentional forms of communication. For some participants, distance unexpectedly improved parent-child relationships by shifting interactions away from daily tensions toward emotional support and mutual appreciation. These findings suggest that geographical separation was not experienced solely as loss, but also as a relational condition that could reshape family dynamics in psychologically meaningful ways.

### Theme 2: moral tension and emotional duality

#### Subtheme 2.1 persistent guilt and moral inadequacy

Many participants expressed feeling “pulled apart” between the weight of traditional norms and the practical constraints of living abroad. Even when they demonstrated considerable competence in coordinating care from afar, including managing medical needs, monitoring parents’ well-being online, and providing steady emotional support through technology, the moral ideal of physical presence remained central to their understanding of what it means to be a good daughter. This value system, shaped by Confucian notions of filial piety, conflicted with the structural conditions of contemporary transnational life. Participants described this conflict as creating a persistent “void” or a “distance that never closes.” One participant captured this tension succinctly:

Sometimes they were telling me how their friend’s daughter or son is fulfilling the filial obligation. They talk about how these sons and daughters take their parents to the park or to travel. You know, they are not blaming me directly, but it is a kind of blame silently. Sometimes, when I can’t sleep at night, or I am taking the subway to work, I just think about the words they said, and I feel like I am not a good daughter.

These feelings of inadequacy persisted even among participants who demonstrated substantial caregiving involvement from abroad, suggesting that emotional distress was not necessarily reduced through increased caregiving competence. Instead, participants appeared to evaluate themselves against culturally internalized ideals of physical co-presence and direct caregiving. As a result, the emotional burden of caregiving was shaped not only by practical caregiving responsibilities, but also by moral expectations surrounding what constitutes a “good daughter” within traditional Chinese familial culture.

#### Subtheme 2.2 migration as both achievement and moral conflict

Other participants described a persistent tension between pursuing personal autonomy abroad and fulfilling filial expectations rooted in Chinese cultural norms. For these individuals, migration was both liberating and morally complex. Although living overseas created opportunities for professional growth, financial stability, and self-realization, it also heightened their awareness of falling short of traditional family ideals. Many characterized their decision to leave home as an experience with two sides: a source of pride and unease, progress and loss. The act of migrating carried existential weight, representing both personal achievement and a perceived departure from filial duty. As one participant reflected,

‘父母在, 不远游’(when the parents are alive, the child shouldn’t go far). I have a better income and lifestyle here, but I also feel bad leaving them alone in China.

Migration was simultaneously associated with independence, opportunity, and upward mobility, while also carrying emotional meanings related to separation, perceived abandonment, and moral compromise. Rather than resolving these tensions, many participants appeared to continuously live within them, suggesting that transnational caregiving involved an enduring coexistence of gratitude, ambition, guilt, and loss.

#### Subtheme 2.3 communication, blame, and relational strain

Participants described communication with parents as a key site where emotional strain is both expressed and intensified. Interactions often shifted between subtle comparison, indirect blame, and protective silence. Participant 1 explained this dilemma clearly:

She only tells me about it afterward because she doesn’t want to trouble me… but that makes me worry even more.

Communication was often characterized by emotional ambivalence, where both parents and daughters attempted to protect one another from distress while unintentionally intensifying relational anxiety. Participants described cycles of concealment, indirect blame, and emotional restraint that reflected broader cultural dynamics surrounding emotional expression, sacrifice, and familial obligation. As a result, communication became not merely an exchange of information, but a site where care, guilt, protection, and emotional tension were continuously negotiated.

### Theme 3: navigating uncertainty and change

#### Subtheme 3.1 living with persistent uncertainty and anticipation

As their parents aged, many participants described living with a growing sense of uncertainty and unease about the future. They were acutely aware that time with their parents was limited, yet they remained unable to close the distance that separated them. One participant stated:

You know, my parents’ health is so unpredictable. They can seem completely fine now, but tomorrow it could be something serious, like cancer. And with everything going on, political tensions, sudden travel restrictions, you never really know if you’ll even be able to go back when something happens.It just makes everything feel so fragile, like it could fall apart at any moment.

The unpredictability of parental aging, combined with immigration policies, geopolitical instability, and travel barriers, contributed to a persistent anticipatory state in which participants remained emotionally prepared for potential crisis. This ongoing vigilance appeared to reshape participants’ emotional lives, future planning, and sense of security.

#### Subtheme 3.2 anticipatory grief and emotional pre-loss

For participants whose parents were already managing chronic illnesses, uncertainty became a constant emotional backdrop. They spoke of the quiet anxiety of not knowing when a condition might worsen or whether they would be able to return home quickly enough in the event of an emergency. Illness was experienced not as a predictable medical course but as a gradual erosion of stability, where each new symptom or hospitalization deepened their awareness of what could soon be lost. One participant stated:

My father’s condition is stable most days, but, you know, every time when the phone rings, I panic. I’m scared it is the bad news, you know, something, like ‘your father’s situation is worsened.’

Several caregivers reflected that the most challenging part of transnational caregiving was not the physical or financial labor, but the uncertainty itself, the daily waiting, the inability to predict what might happen next. This emotional weight was intensified by distance; they could send money, coordinate doctors, and manage appointments online, yet still feel powerless in the moments that mattered most.

Moreover, for many participants, remote caregiving involved a quiet rehearsal of loss. Even before death occurred, they experienced waves of anticipatory grief, moments of sadness, guilt, and fear that surfaced whenever they thought about their parents’ aging bodies or imagined their final days. A few participants had already lived through this loss.

If my mom lives another five years… that’s less than a hundred days together. Whenever I think about it, I just cry.

Over time, many participants described a gradual integration of loss and uncertainty into their broader understanding of life. Rather than resisting the emotional strain of distance, they began to accept it as part of their lived reality—a condition to live with rather than to overcome. This acceptance did not mean resignation; instead, it reflected a quiet adaptation to the enduring ambiguities of transnational caregiving.

#### Subtheme 3.3 life adjustment

Several participants also made practical life adjustments to reduce distance. They reported relocating to cities closer to their parents, changing jobs to allow flexible travel, or modifying citizenship status to facilitate long-term caregiving plans remotely. For example, one participant mentioned “I gave up my Chinese passport to become an American citizen; it is not an easy decision, you know, I lose some benefits. But, you know, my parents could get a green card.”

These adjustments illustrate how caregiving responsibilities extended beyond emotional support into long-term life restructuring. Participants described modifying career trajectories, migration plans, citizenship decisions, and lifestyle arrangements in response to anticipated caregiving demands. Such decisions demonstrate that transnational caregiving was not experienced as a peripheral responsibility, but as a central organizing force shaping participants’ identities, future orientations, and major life choices.

## Discussion

As one of the few exploratory studies in this field, this research contributes to the growing body of literature on how women from China’s one-child generation navigate long-distance caregiving in transnational contexts. Rather than viewing caregiving through the lens of logistics or physical distance, these findings frame it as an active interpretive process shaped by emotional negotiation, cultural expectations, and shifting relational dynamics. Ultimately, caregiving across borders functions as a redefinition of presence and responsibility within a framework of significant structural constraints.

### Key finding 1: internalized moral strain in transnational caregiving

A central finding of this study is that transnational caregiving was experienced as a form of internalized moral strain, in which structural barriers were frequently interpreted as personal inadequacy. Participants often described feelings of guilt when they perceived themselves as unable to fulfill filial expectations despite constraints imposed by immigration policies, financial limitations, and geographic distance. In psychological literature, guilt is understood as an emotional response to perceived wrongdoing, whereas blame reflects the attribution of responsibility for negative outcomes [14]. Rather than attributing caregiving difficulties to external structural conditions, many participants internalized these limitations as individual failure, intensifying emotional distress and negatively shaping caregiver identity.

This emotional burden was further reinforced through relational dynamics within the family. Participants frequently interpreted parental dissatisfaction, emotional restraint, or limited acknowledgment of caregiving efforts as implicit judgment, which deepened feelings of inadequacy and lowered self-worth. These findings align with prior research suggesting that sustained criticism or perceived disapproval can negatively influence self-esteem and psychological well-being [6]. In response, some caregivers described emotionally withdrawing or reducing communication as a protective strategy under prolonged emotional strain, reflecting patterns of avoidance commonly observed in emotionally overloaded caregiving relationships [17].

At the same time, parents’ reluctance to disclose illness, distress, or vulnerability intensified caregivers’ feelings of helplessness and anxiety. Participants frequently interpreted this limited disclosure as shaped by cultural norms discouraging emotional expression among older Chinese adults and by expectations that adult children should remain primary sources of support regardless of geographic distance. These findings are consistent with research demonstrating that stigma surrounding vulnerability and emotional disclosure remains prevalent among older adults in Chinese cultural contexts [9].

### Key finding 2: negotiating dual commitments between personal aspiration and filial responsibility

A second key finding highlights that transnational caregiving involves an ongoing negotiation between personal aspirations and culturally embedded filial obligations. Participants frequently described feeling gratitude for the educational or professional opportunities enabled by migration while simultaneously feeling guilty for their physical absence. Cultural norms, reinforced through proverbs such as “父母在, 不远游” (while parents are alive, one should not travel far), remained salient moral reference points, shaping participants’ perceptions of what constitutes appropriate filial conduct.

These findings reflect the dual attachments characteristic of transnational family life, in which individual mobility coexists with enduring intergenerational responsibility [36]. Although migration disrupted traditional caregiving arrangements such as co-residence and direct daily care [19], these expectations continued to influence how participants interpreted their caregiving roles and responsibilities. Rather than resolving the tension between personal aspiration and filial obligation, caregivers continuously redefined how caregiving could be enacted across distance under structural constraints.

Importantly, this process was not solely experienced as conflict. Many participants also identified positive transformations, including increased self-efficacy, emotional maturity, and strengthened relationships with their parents. Expressions of gratitude and reciprocal acknowledgment played a critical role in sustaining these positive meanings, reinforcing caregivers’ sense of purpose and competence [32]. Even across distance, emotionally meaningful exchanges fostered a sense of closeness that counterbalanced separation, consistent with affective theories of social exchange [12].

### Key finding 3: living with anticipation

A particularly important contribution of this study is the identification of caregiving as a future-oriented emotional and interpretive process. While caregiving research has traditionally focused on present caregiving responsibilities, participants in this study described caregiving as equally shaped by anticipation, uncertainty, and preparation for future parental decline. Their narratives suggest that caregiving extended beyond immediate caregiving tasks and became embedded within long-term emotional planning and future-oriented meaning-making.

Persistent uncertainty emerged as a defining feature of participants’ experiences. Anxiety surrounding parental health was intensified by geographic separation, limited communication, and difficulty obtaining timely information regarding medical conditions or daily living needs. These findings are consistent with Mishel [28] Uncertainty in Illness Theory, which proposes that ambiguity and insufficient information contribute significantly to psychological distress. Similar patterns have been observed among caregivers managing uncertainty in chronic illness contexts [16]. Participants further described how geopolitical tensions, travel restrictions, and the lingering effects of the COVID-19 pandemic heightened perceptions of instability and unpredictability surrounding future caregiving plans [34].

Participants’ anticipatory concerns were also shaped by practical challenges that required emotionally significant decisions. Many noted that China’s health insurance system excludes foreign visitors, forcing them to consider difficult alternatives to secure future healthcare for their parents. Some relinquished their Chinese citizenship to obtain U.S. green cards for their parents, while others debated whether to return to China to provide full-time care eventually. These decisions involved interpreting complex legal, financial, and emotional trade-offs, consistent with research findings that bureaucratic constraints intensify caregivers’ psychological burden [1]. Strategic relocation within the United States was another response to anticipated caregiving needs. Several participants considered moving to cities with direct flights to China so they could travel more quickly in emergencies. These considerations illustrated the extent to which caregiving shaped participant’s long-term planning and revealed how responsibility, identity, and mobility were intertwined in their meaning-making.

Anticipatory grief emerged as another significant dimension of participants’ future-oriented experiences. Participants described persistent fear surrounding parental decline and distress associated with the possibility of being absent during critical moments of illness or death. Experiences of losing relatives during the COVID-19 pandemic intensified these fears and contributed to unresolved emotional distress consistent with ambiguous loss, in which grief occurs without clear resolution or closure [5]. Unlike acute grief following bereavement, participants described prolonged emotional vigilance characterized by persistent worry, intrusive thoughts, and imagined future loss. These findings are consistent with both early conceptualizations of grief [25] and more recent evidence demonstrating that prolonged anticipation of loss may generate significant psychological distress, including anxiety, sadness, and emotional exhaustion [26].

Importantly, uncertainty, anticipatory grief, and caregiving responsibility did not emerge as isolated emotional experiences. Rather, these dimensions were deeply interconnected within participants’ future-oriented meaning-making processes. Caregivers continuously evaluated their emotional and practical capacity to respond to future caregiving demands while attempting to maintain personal, professional, and transnational lives. This anticipatory orientation represents an underexamined dimension of transnational caregiving and suggests the need for future research examining how uncertainty, migration, and culturally embedded filial expectations interact to shape long-term caregiver psychological well-being.

Across all narratives, caregivers made meaning by imagining future caregiving scenarios and evaluating their capacity to meet anticipated challenges. Their reflections showed that caregiving was not only a response to immediate needs but also a forward-looking process influenced by uncertainty, anticipated loss, cultural expectations, and emotional responsibility. This anticipatory orientation represents a critical, underexamined dimension of transnational caregiving, highlighting how caregivers construct meaning as they prepare for futures marked by unpredictability and obligation.

### Implication

Theoretically, this study contributes to caregiving scholarship by illustrating how cultural expectations, migration-related constraints, and interpersonal dynamics jointly shape caregiving experiences in transnational contexts. The findings further suggest that caregiving cannot be understood solely through binary frameworks of burden versus reward, as participants simultaneously described emotional strain, responsibility, resilience, and relational closeness. In addition, the findings highlight the importance of future-oriented caregiving concerns, including ongoing preparation for potential parental decline, uncertainty surrounding future care needs, and emotional responses associated with anticipated separation and loss. While anticipatory dimensions of caregiving and grief have been discussed in prior literature, this study suggests that these experiences may take distinct forms within long-distance transnational caregiving and therefore warrant further conceptual and empirical examination.

Given that participants are predominantly women, the findings have clear implications for women’s health. The cumulative demands of transnational caregiving, marked by distance, uncertainty, and competing roles, create a chronic stress context that may increase risks of anxiety, depressive symptoms, sleep disturbance, and burnout [35]. Prolonged strain may also contribute to fatigue and hormonal disruptions, which are particularly relevant to women’s health across the life course [15]. Accordingly, multi-level interventions are needed. Clinically, providers should incorporate routine screening for caregiver burden, mental health, and sleep, while encouraging preventive care engagement.

### Limitation

This study has several limitations. First, although it focuses on married only-child daughters to ensure analytical specificity, it does not systematically examine how marital status shapes migration-related constraints, time allocation, and emotional burden. Future research should explore these dimensions more directly. Second, recruitment through social media and snowball sampling may introduce sampling bias and limit sample diversity, potentially affecting the transferability of the findings. More diverse recruitment strategies are needed in future studies. Third, as the study relies on self-reported interview data, the findings may be influenced by recall and social desirability biases. Future research could incorporate multiple data sources or longitudinal designs to strengthen validity.

## Supplementary Information

Supplementary Material 1.

## Data Availability

The datasets generated and/or analyzed during the current study are not publicly available due to ethical and privacy considerations but are available from the corresponding author upon reasonable request.
