# Supplementary material for: The lived experiences of female only-child caregivers in transnational settings
Source: BMC Womens Health. 2026 May 25;26:371. doi: 10.1186/s12905-026-04548-2 (PMC13386826; doi:10.1186/s12905-026-04548-2)
Supplement: Supplementary file 1 — Supplementary Material 1. [file 12905_2026_4548_MOESM1_ESM.docx]

**Appendix A: Interview protocol**

**Section 1: Basic Background Information**

1. How many parent(s) are you currently caring for? (Include parent(s)-in-law if

applicable)

2. How old is each of your parents?

3. In which city are your parents currently living?

4. Do your parents live by themselves or with others?

5. Is there anyone currently hired to assist your parents?

6, How long have you been in a caregiving role for your parent(s)?

7. What is your approximate annual income?

8. What is your age?

9. In which city do you currently reside?

**Section 2: Overall Caregiving experiences**

1. If you had to use one word to describe your experience caring for your parents, what word would you choose? Can you elaborate?

**a.** Can you share a rewarding moment in your caregiving experience?

**b.** Can you describe a challenging moment in your caregiving experience?

**c.** Have there been any instances where you had to prioritize caregiving over your personal needs (e.g., traveling, working, socializing, etc.)?

**d.** Have this caregiving experiences changed your understanding of yourself or your relationship with your parents?

**e.** How have these experiences influenced your approach to caregiving moving forward?

**Section 3: Caring from Distance**

**Do you perceive distance caregiving as offering more benefits or more burdens?** **Please elaborate.**

**a.**What activities do you perform to care for your parents from afar?

• Have these activities changed over time?

**b.** How do you feel about not being able to care for your parents physically?

**c.** Have you made any compromises to care for your parent(s) from afar? (e.g., probe: vacation, resting time, socialization, other?)

**Section 4: How has the principle of filial piety (Xiao) influenced your caregiving** **approach?**

**a.** How did you learn about these traditions or values?

**b.** Are any values besides filial piety guiding you in caring for your parents?

**c.** Have any of these values changed over time?

ii. Have you noticed any situations (such as moving abroad, marriage, or having children) that prompted you to

reshape your caregiving values?

**d.** Do you find that filial Piety supports your caregiving role, or does it add to your caregiving burden? Please elaborate.
